# Impairment of Serotonergic Transmission by the Antiparkinsonian Drug L-DOPA: Mechanisms and Clinical Implications

**DOI:** 10.3389/fncel.2017.00274

**Published:** 2017-09-12

**Authors:** Cristina Miguelez, Abdelhamid Benazzouz, Luisa Ugedo, Philippe De Deurwaerdère

**Affiliations:** ^1^Department of Pharmacology, Faculty of Medicine and Dentistry, University of the Basque Country (UPV/EHU) Leioa, Spain; ^2^Institut des Maladies Neurodégénératives, Université de Bordeaux, UMR 5293 Bordeaux, France; ^3^CNRS, Institut des Maladies Neurodégénératives, UMR 5293 Bordeaux, France

**Keywords:** serotonin, dopamine, electrophysiology, intracerebral microdialysis, depression, dyskinesia, Parkinson’s disease, exocytosis

## Abstract

The link between the anti-Parkinsonian drug L-3,4-dihydroxyphenylalanine (L-DOPA) and the serotonergic (5-HT) system has been long established and has received increased attention during the last decade. Most studies have focused on the fact that L-DOPA can be transformed into dopamine (DA) and released from 5-HT terminals, which is especially important for the management of L-DOPA-induced dyskinesia. In patients, treatment using L-DOPA also impacts 5-HT neurotransmission; however, few studies have investigated the mechanisms of this effect. The purpose of this review is to summarize the electrophysiological and neurochemical data concerning the effects of L-DOPA on 5-HT cell function. This review will argue that L-DOPA disrupts the link between the electrical activity of 5-HT neurons and 5-HT release as well as that between 5-HT release and extracellular 5-HT levels. These effects are caused by the actions of L-DOPA and DA in 5-HT neurons, which affect 5-HT neurotransmission from the biosynthesis of 5-HT to the impairment of the 5-HT transporter. The interaction between L-DOPA and 5-HT transmission is especially relevant in those Parkinson’s disease (PD) patients that suffer dyskinesia, comorbid anxiety or depression, since the efficacy of antidepressants or 5-HT compounds may be affected.

## Introduction

Parkinson’s disease (PD) is a neurodegenerative disorder characterized by motor symptoms such as bradykinesia, rigidity, resting tremor, postural abnormalities and gait deficits. However, most patients also develop additional non-motor symptoms, such as anxiety, depression, fatigue, apathy, mild cognitive disturbances and dementia (Bastide et al., 2015). The disease is no longer seen as a specific consequence of dopaminergic (DA) neurodegeneration, since other neuronal systems, such as the noradrenergic and serotonergic (5-HT) systems, also suffer alterations in the course of the disease (Jenner et al., 1983; Delaville et al., 2011). The 5-HT system is involved in some PD pathological clinical manifestations as well as in some side effects induced by long-term treatment using L-3,4-dihydroxyphenylalanine (L-DOPA), such as L-DOPA-induced dyskinesia (Scholtissen et al., 2006a,b; Haduch et al., 2016). It has been suggested that 5-HT neurons are mainly responsible for the increase in DA release induced by chronic L-DOPA treatment in the striatum (Carta et al., 2007, 2008). However, the motor improvements or side effects produced by low doses of L-DOPA (3–6 mg/kg) cannot be explained only by what happens in the striatum since those doses fail to produce physiological levels of DA in that nucleus (Navailles et al., 2010b, 2011; Nevalainen et al., 2011; Porras et al., 2014; De Deurwaerdère et al., 2017). Indeed, L-DOPA may act through other mechanisms (Misu et al., 1996; Porras et al., 2014; De Deurwaerdère et al., 2017) or modify DA transmission in other brain regions to a greater extent than in the striatum (Navailles and De Deurwaerdère, 2012b). However, despite the evidence that L-DOPA alters the 5-HT system (Navailles and De Deurwaerdère, 2012a), and that 5-HT receptor agonists can produce therapeutic benefit in L-DOPA-induced dyskinesia and psychosis, the impact of L-DOPA on 5-HT neurotransmission has been poorly studied (De Deurwaerdère et al., 2017).

The purpose of this mini-review is to assess the experimental data highlighting the complex effects of L-DOPA on 5-HT extracellular levels and ultimately on 5-HT transmission.

## Serotonergic Neurons Mediate L-DOPA-Derived DA Release

L-DOPA can be taken up by virtually all cells in the brain and decarboxylated into DA in cells expressing amino acid decarboxylase (AADC) and other types of decarboxylase such as histidine decarboxylase (De Deurwaerdère et al., 2017). The reason why 5-HT neurons differ from other cell types expressing AADC is that in 5-HT neurons newly synthesized DA can directly compete with 5-HT for inclusion in exocytotic vesicles via the vesicular monoamine transporter VMAT2 (Lohoff, 2010; De Deurwaerdère et al., 2017). L-DOPA-induced DA is thereby concentrated in 5-HT vesicles, probably at the expense of endogenous 5-HT (see below), and is released by 5-HT neurons in well-innervated areas of the whole brain (Navailles et al., 2010b). However, 5-HT cells lack the mechanisms to control DA release, as 5-HT_1A_ and 5-HT_1B_ autoreceptors and 5-HT transporters (SERT) do not detect extracellular DA. Several microdialysis studies show that L-DOPA, at 12 mg/kg and higher doses, induces excessive DA release (Abercrombie et al., 1990; Kannari et al., 2000; Navailles et al., 2010b). However, the amount of released DA may have been overestimated in animal models of PD, as there are no clearance mechanisms and consequently extracellular L-DOPA-derived DA is detected by probes in significant quantities (Miller and Abercrombie, 1999). In fact, the real levels of extracellular DA in the striatum are probably much lower than those measured, given the low density of 5-HT terminals in the striatum (De Deurwaerdère et al., 2017).

In general, 5-HT neurons have the required enzymatic/transporter equipment to transform L-DOPA into DA and release it. However, 5-HT neurons lack the proper autoregulatory mechanisms to control DA release and clearance, which can cause excessive DA levels in several brain regions.

## Effect of L-DOPA on the Electrical Activity of 5-HT Neurons

Several studies suggest that L-DOPA could modify the activity of 5-HT neurons. First, DA agonists increase dorsal raphe nucleus (DRN) activity in control animals (Martín-Ruiz et al., 2001); second, DA neurons degeneration unevenly alters the electrophysiological characteristic of DRN neurons (Zhang et al., 2007; Guiard et al., 2008; Kaya et al., 2008; Wang et al., 2009; Prinz et al., 2013) and third, DA and 5-HT release from 5-HT cell bodies and terminals may also impact 5-HT system activity. A computational study has modeled the latter impact, which predicts that L-DOPA administration will decrease 5-HT release in the DRN and subsequently increase DRN neuron firing rate (Reed et al., 2012).

Patch clamp recordings performed in brain slices from control animals with intact DA innervation demonstrated that acute L-DOPA depresses 5-HT_1A_ receptor-mediated transmission in the DRN (Gantz et al., 2015) and discretely increases the basal firing activity of 5-HT neurons (Prinz et al., 2013). On the other hand, in a study performed in anesthetized rats, systemic administration of therapeutic doses of L-DOPA did not modify DRN activity (Miguelez et al., 2013).

In animal models of PD, neither acute nor chronic systemic administration of L-DOPA altered the neuronal activity of DRN cells (Miguelez et al., 2016a,b). *In vitro*, L-DOPA partially reversed the changes in excitability observed in Parkinsonian mice (Prinz et al., 2013).

In conclusion, the effect of L-DOPA on DRN neuron activity seems non-existent or discrete, suggesting that the interaction between the 5-HT system and L-DOPA may be more relevant at the terminal rather than the somatic level.

## Effect of L-DOPA on 5-HT Extracellular Levels

**Figure 1:**
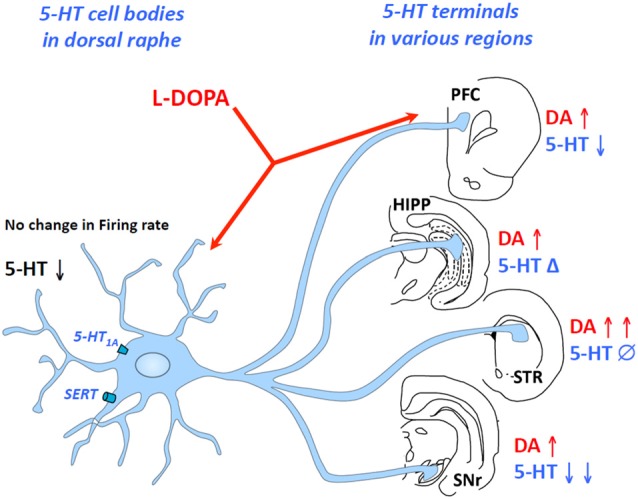
Region-dependent effects of L-3,4-dihydroxyphenylalanine (L-DOPA) on serotonin (5-HT) extracellular levels in some brain regions. L-DOPA acts at the level of 5-HT cell bodies in the dorsal raphe nucleus (DRN) and 5-HT terminals in the brain including the prefrontal cortex, the hippocampus, the striatum or the substantia nigra pars reticulate. While L-DOPA triggers an increase in dopamine (DA) release in all regions, it inhibits 5-HT release in the substantia nigra reticulata, the prefrontal cortex and presumably in the DRN, induces biphasic effects (Δ) in the hippocampus and merely affects 5-HT release in the striatum. The changes in DA and 5-HT extracellular levels occur without any modification of 5-HT neuron activity. HIPP, hippocampus; PFC, prefrontal cortex; STR, striatum; SNr, substantia nigra reticulate.

As discussed above, L-DOPA-derived DA competes with 5-HT to enter exocytotic vesicles at 5-HT terminals, which likely leads to an inhibition of 5-HT release. However, this hypothesis has not been fully supported by *in vitro* and *in vivo* data, which suggests that the effects of L-DOPA on extracellular 5-HT levels are more complex than previously thought. Indeed, when applied locally, L-DOPA enhances the 5-HT efflux (Biggs and Starr, 1999) while when administered systemically, it either does not alter or reduces 5-HT extracellular levels (Lindgren et al., 2010; Navailles et al., 2010a, 2014; Navailles and De Deurwaerdère, 2012a). Furthermore, L-DOPA’s effects on extracellular 5-HT levels are not similar in all regions innervated by the DRN including no effect (striatum), inhibition (substantia nigra and cortex) and biphasic excitation/inhibition (hippocampus; Navailles et al., 2011; Navailles and De Deurwaerdère, 2012b; Figure 1).

In accordance with the idea that newly synthesized DA displaces 5-HT inside exocytotic vesicles, several publications show that acute administration of L-DOPA reduces 5-HT tissue content, which mainly represents 5-HT stored in vesicle compartments, or its metabolite 5-hydroxyindolacetic acid (Eskow Jaunarajs et al., 2012; Miguelez et al., 2016b). Nonetheless, some discrepancies exist which could depend on the doses used of L-DOPA or AADC inhibitor, or the time of sacrifice after L-DOPA administration. After chronic administration of L-DOPA (12 mg/kg), L-DOPA further inhibited extracellular 5-HT levels in the hippocampus (amplified the inhibitory component of the biphasic effect) and substantia nigra, while its effect was unchanged in the cortex and the striatum (Navailles et al., 2011). In control and Parkinsonian monkeys, the acute administration of L-DOPA reduces 5-HT tissue levels in the striatum and motor cortex. In contrast, chronic L-DOPA treatment reduced 5-HT levels in the striatum, hippocampus or amygdala of Parkinsonian but not control monkeys (Engeln et al., 2015). In Parkinsonian rats, chronic treatment using 6 mg/kg of L-DOPA reduces 5-HT content in a region-dependent manner (Stansley and Yamamoto, 2014, 2015a).

In summary, although local administration of L-DOPA may increase 5-HT levels, systemic administration reduces it in several brain regions without modifying DRN neuron activity and modestly reducing 5-HT extracellular levels.

## The Role of Non-Exocytotic Mechanisms in the Effect of L-DOPA

As anticipated as early as 1970 (Ng et al., 1970), apart from exocytotic release, L-DOPA also triggers non-exocytotic (non-vesicular) efflux of 5-HT and DA. Indeed, supra-therapeutic dose [100 mg/kg (De Deurwaerdère et al., 2017)] of L-DOPA transiently enhances 5-HT release. A Ca^2+^ free medium magnified this effect and unmasked excitation of 5-HT release induced by a therapeutic dose (12 mg/kg) of L-DOPA (Miguelez et al., 2016b). In addition, blockade of action potential-dependent presynaptic release or Ca^2+^ removal in the perfusion solution does not suppress L-DOPA-induced DA release (Miller and Abercrombie, 1999; Lindgren et al., 2010; Miguelez et al., 2016b).

**Figure 2:**
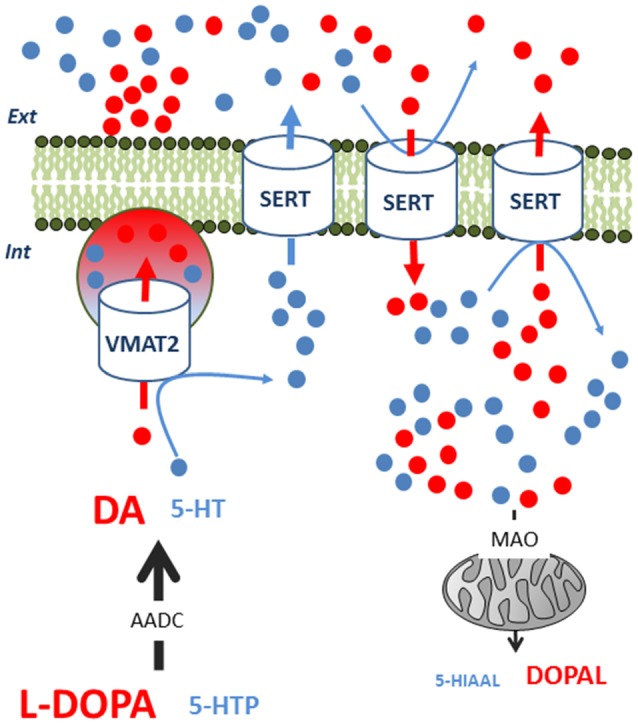
Competition between L-DOPA-derived dopamine (DA) and serotonin (5-HT) inside 5-HT neurons. L-DOPA competes with 5-HTP for AADC to synthesize DA and 5-HT, respectively. DA competes with 5-HT in terms of VMAT2-mediated packaging of exocytotic vesicles. In consequence, intracellular 5-HT levels can transiently rise and 5-HT can exit the neuron via SERT in a non-exocytotic manner. This 5-HT output can only be observed under specific conditions, as L-DOPA-derived DA can also alter the function of SERT. First, extracellular DA can undergo reuptake by SERT, reducing the 5-HT flow through this transporter. Second, intracellular DA can also enter the neuron through the SERT, impairing the output of 5-HT. In the cytoplasm, MAO can more efficiently degrade DA than 5-HT, increasing oxidative metabolism and aldehyde derivates. These biochemical events occur with no modification of the firing rate of 5-HT neurons. AADC, amino acid decarboxylase, L-DOPA, 3,4-Dihydroxyphenylacetaldehyde; MAO, monoamine oxidase; SERT, serotonin transporter; VMAT2, monoamine vesicular transporter; 5-HIAAL, 5-hydroxyindole acetaldehyde; 5-HTP, 5-hydroxytryptophan.

The mechanism underlying the non-exocytotic release of neurotransmitters is still unclear. It may also require 5-HT neurons (Tanaka et al., 1999; Navailles et al., 2010b) and the involvement of one or several transporters, but the effect also shows region dependency. Indeed, SERT blockade by citalopram significantly reduced peripheral L-DOPA-induced DA release in the hippocampus but not in the prefrontal cortex (PFC; Miguelez et al., 2016b; Figure 2).

The participation of a non-vesicular release of both DA and 5-HT in different brain regions may contribute to the heterogeneous effect of L-DOPA on these neurotransmitters. The whole biochemical picture appears to be particularly complicated for 5-HT as it is displaced by DA in exocytotic vesicles, trapped in the neuron by its reduced ability to exit via the SERT, and is perhaps more available to degradation by monoamine oxidase (Figure 2; Stansley and Yamamoto, 2014). Moreover, the metabolism of both DA and 5-HT could have detrimental effects on the survival of groups of 5-HT neurons (Navailles et al., 2011; Stansley and Yamamoto, 2015a). Indeed, L-DOPA can promote oxidative stress and generate free radicals or toxic compounds. Therefore, L-DOPA-derived DA can be metabolized into pro-oxidant quinones, 3,4-dihydroxyphenyacetic acid or hydrogen peroxide and produce cell death in the DRN (Stansley and Yamamoto, 2015b).

To sum up, *in vitro* experiments and mathematical models predict L-DOPA function in 5-HT terminals (Reed et al., 2012) but none of these models fully describe the region dependency and the complex modalities of release induced by L-DOPA *in vivo*.

## Altered Reactivity of 5-HT Neurons in the Presence of L-DOPA

After chronic treatment using L-DOPA and/or DA denervation, the inhibitory control exerted by 5-HT_1A_ receptors over the electrical activity of 5-HT neurons is maintained (Miguelez et al., 2016a). In these conditions, 5-HT_1A_ receptor agonists completely inhibit DRN neuron activity and diminish L-DOPA-induced DA release at the terminal sites (Kannari et al., 2001; Iderberg et al., 2015). However, while the firing rate is completely suppressed, DA release still occurs. This result is compatible with the evidence that some mechanisms related to DA release are independent of the electrical activity of 5-HT neurons. The control exerted by 5-HT_1B_ receptors at the terminals might not be fully functional by itself because the stimulation of these receptors alone does not reduce L-DOPA-stimulated striatal DA release (Kannari et al., 2001) but ameliorates dyskinesia when co-administered together with 5-HT_1A_ agonists (Muñoz et al., 2009). The activation of other 5-HT receptors can also modulate L-DOPA-stimulated DA release. In this regard, 5-HT_4_ receptor stimulation can indirectly activate the activity of 5-HT neurons (Ge and Barnes, 1996; Lucas and Debonnel, 2002; Lucas et al., 2005, 2007) and enhance L-DOPA-induced DA release in the PFC and the substantia nigra, but not in the hippocampus or the striatum (Navailles et al., 2015).

In Parkinsonian conditions, SERT density might not be altered but L-DOPA-derived DA can directly and indirectly modify SERT function. As reported above, DA can also bind to SERT and compete with 5-HT for non-exocytotic efflux through this transporter (Figure 2). The indirect impairment is related to a possible decrease in 5-HT cellular levels. Indeed, fluoxetine or citalopram can reduce L-DOPA-stimulated DA release (Yamato et al., 2001; Navailles et al., 2010b) through a mechanism partly involving 5-HT_1A_ receptors (Yamato et al., 2001). However, the efficacy of fluoxetine to inhibit the electrical activity of 5-HT neurons in the DRN can be reduced by L-DOPA, in line with a possible inhibitory effect of L-DOPA on extracellular levels of 5-HT (Miguelez et al., 2016a). Behavioral studies support a lower efficacy of selective serotonin reuptake inhibitors (SSRIs) in the presence of L-DOPA (Miguelez et al., 2013; Fidalgo et al., 2015). Because the SERT plays a critical role in the various outcomes of L-DOPA-induced changes of DA and 5-HT extracellular levels, its indirect modulation by 5-HT_2B_ receptor ligands can be tested. Indeed, 5-HT_2B_ receptors can be considered as autoreceptors involved in the control of SERT activity and their antagonism has been shown to block outward release induced by the 5-HT/DA releaser 3,4-methylenedioxymethamphetamine (Gudelsky and Yamamoto, 2008).

Extracellular levels of 5-HT in the presence of L-DOPA can be impacted in different ways by: (1) decreased exocytosis, which non-exocytotic release can compensate for to a limited extent; (2) the loss of extracellular clearance by the SERT (competition with DA); and (3) the loss of other clearance mechanisms such as the noradrenaline transporter (Navailles et al., 2014). On the basis of the described data, it seems that extracellular levels of 5-HT are almost impossible to control in a context where all these factors are region dependent. For instance, the inhibition of levels of extracellular 5-HT induced by the high frequency stimulation of the subthalamic nucleus was attenuated by the presence of L-DOPA (technically an increase compared with what was expected), while L-DOPA-stimulated DA extracellular levels were partly decreased (Navailles et al., 2010a). L-DOPA disrupts the control of extracellular 5-HT levels, and the biological function of this phenomenon remains completely unknown.

In summary, the release of L-DOPA-derived DA from 5-HT neurons is a complicated mechanism involving 5-HT receptors and transporters in a region-dependent manner.

## From the Neurochemistry to the Clinic

The side effects induced by L-DOPA are usually interpreted as a consequence of the modification of L-DOPA-stimulated DA release from 5-HT neurons involving alteration of 5-HT neurotransmission. Although the published results are heterogeneous, it is accepted that L-DOPA induces adaptive and/or toxic changes in the 5-HT system that may have clinical relevance. One study in postmortem tissue described a positive correlation between SERT binding densities and the development of dyskinesia (Rylander et al., 2010); however, subsequent studies in postmortem samples or patients did not find any correlation between 5-HT or SERT in the striatum and dyskinesia (Politis et al., 2014; Cheshire et al., 2015). These discrepant results may be related to the overestimated role of the striatum in dyskinesia (De Deurwaerdère et al., 2017). Indeed, several brain regions other than the striatum have been proposed to participate in dyskinesia. A recent publication showed that SERT binding was positively correlated with the manifestation of dyskinesia in the internal and external parts of the globus pallidus (Smith et al., 2015). Regarding 5-HT receptors, PD is marked by the uneven modification of some 5-HT receptor subtypes in different brain regions. Therefore, Parkinsonian patients treated using L-DOPA show higher expression of 5-HT_1A_ and 5-HT_2C_ receptors in the cerebral cortex, but not in other brain regions (Huot et al., 2010, 2012).

The clinical implications of these 5-HT adaptive changes could underlie the efficacy of drugs acting on 5-HT neurotransmission to reduce the severity of dyskinesia. Although in animal models of the disease several drugs acting on 5-HT_1A/1C_ or 5-HT_1B_ receptors decrease the expression of L-DOPA-induced dyskinesia (Miguelez et al., 2013), translational extrapolation to the clinic has been less positive. So far, only two drugs have been proven to be clinically efficient in treating dyskinesia. These are eltoprazine, a 5-HT_1A/B_ receptor agonist (Bomasang-Layno et al., 2015; Svenningsson et al., 2015), and buspirone, a partial agonist of the 5-HT_1A_ receptor, which discretely ameliorates dyskinesia (Politis et al., 2014). In general, 5-HT compounds have been demonstrated to be less efficacious than expected, probably because the existing theories about 5-HT and DA interaction, especially in the striatum, are too simplistic.

Another important clinical aspect to take into account is the fact that often antidepressants and L-DOPA are administered together. Commonly used SSRIs enhance 5-HT transmission in depressed patients; however, in PD the DRN undergoes degeneration contributing to worse therapeutic control of depressive symptoms in these patients (Deurwaerdère and Ding, 2016). Preclinical data show a loss of efficacy of SSRIs and suggest the use of other strategies such as noradrenaline uptake inhibitors (Miguelez et al., 2013). Unfortunately, the clinical data are unclear. Some studies suggest that SSRIs are less efficacious for treating depression in PD and should be used only as a last choice (Aarsland et al., 2009; Skapinakis et al., 2010; Liu et al., 2013; Rocha et al., 2013). However, a recent meta-analysis reports that treatment using SSRIs significantly improves depression among PD patients (Bomasang-Layno et al., 2015). Other antidepressants, such as the mixed noradrenaline and 5-HT uptake inhibitor, venlafaxine, had similar efficacy as the SSRI paroxetine (Richard et al., 2012; Broen et al., 2016). However, more studies should be conducted to verify which antidepressant has a better therapeutic profile when specifically co-administered with L-DOPA, which is rarely evaluated.

L-DOPA can also produce psychosis in the advanced stages of the PD, although less frequency than dyskinesia. The manifestation of psychosis could depend on an excess of DA transmission, presumably in cortical areas (De Deurwaerdère and Di Giovanni, 2017), and drugs that limit the excess of DA release from 5-HT neurons could be therapeutically interesting. Therefore, the atypical antipsychotic drugs clozapine, risperidone and olanzapine that interact with 5-HT_1A_, 5-HT_2A_ and 5-HT_2C_ receptors are efficacious even at doses lower than those classically used in the treatment of schizophrenia. The 5-HT_2A_ inverse agonist primavanserin, recently approved to treat PD psychosis, has shown discrete therapeutical improvements in clinical trials although more postmarketing studies are necessary (Meltzer et al., 2010; Cummings et al., 2014; Divac et al., 2016). In terms of mechanism of action, it is unclear whether primavanserin reduces 5-HT tone at 5-HT_2A_ receptors and/or stabilizes DA transmission.

## Conclusion

The effects of L-DOPA on both DA and 5-HT extracellular levels involve 5-HT neurons. These effects are complex and involve several mechanisms. One of the most surprising outcomes is the region-dependent effect for both neurotransmitters. Regarding 5-HT levels, there is a disparity between the results from electrophysiological, biochemical and neurochemical studies. L-DOPA affects 5-HT neurotransmission in the brain through the numerous actions of L-DOPA and DA inside 5-HT neurons, i.e., effects on 5-HT biosynthesis or on 5-HT transporter level and neuron survival. Importantly, the impact of L-DOPA on 5-HT transmission concerns the therapeutic effects of 5-HT drugs on L-DOPA-induced dyskinesia, comorbid anxiety and depression in PD patients.

## Author Contributions

CM and PDD have proposed the architecture of the article and proposed the draft that was implemented by LU and AB. CM and PDD designed the figure and edited the manuscript.

## Conflict of Interest Statement

The authors declare that the research was conducted in the absence of any commercial or financial relationships that could be construed as a potential conflict of interest.

## Abbreviations

5-HT: serotonin
5-HTTP: 5-hydroxytryptophan
5-HIAAL: 5-hydroxyindole acetaldehyde
AADC: amino acid decarboxylase
DA: dopamine
DRN: dorsal raphe nucleus
HIPP: hippocampus
L-DOPA: L-3,4-dihydroxyphenylalanine
MAO: monoamine oxidase
PD: Parkinson’s disease
PFC: prefrontal cortex
SERT: serotonin transporter
SNr: substantia nigra reticulate
SSRI: selective serotonin reuptake inhibitors
STR: striatum
VMAT2: monoamine vesicular transporter.
